# Public and dental professionals' use of social media to discuss amelogenesis imperfecta

**DOI:** 10.1111/ipd.13015

**Published:** 2022-07-18

**Authors:** Victoria Henrick, Samantha Marks, Richard Balmer, Sophy Barber

**Affiliations:** ^1^ School of Dentistry University of Leeds Leeds UK; ^2^ University of Leeds Leeds UK; ^3^ Mid Yorkshire NHS Hospitals Trust Wakefield UK

**Keywords:** amelogenesis imperfecta, dentistry in social media, experience, quality of life, social media

## Abstract

**Background:**

Amelogenesis imperfecta (AI) is an inherited disorder of enamel development that is challenging to treat and often associated with negative patient and parental outcomes. Social media provides a valuable perspective on patients' and dental professionals' experience of AI and dental care.

**Aim:**

To explore how the public and dental professionals use social media to discuss AI.

**Design:**

A cross‐sectional study involving a systemic search of eight social media platforms using the search term ‘amelogenesis imperfecta’. Relevant posts were selected using predefined eligibility criteria. Word content of eligible posts was qualitatively analysed using a thematic framework approach.

**Results:**

A total of 555 posts were identified, of which 144 were eligible for analysis. For dental professionals, the posts included case reports and seeking and sharing of information. For the public, the posts were related to individuals' experience of AI, dental treatment and outcome of treatment.

**Conclusions:**

Posts from individuals affected by AI suggest a need for better distribution of reliable information and greater support. Case reports indicate that dental professionals find it challenging to recognise AI and determine appropriate treatment options. Social media could potentially be used to inform and support people with AI and allow dental professionals to share information and learning with peers.

**Why this paper is important to paediatric dentists**

- Amelogenesis imperfecta is a condition with significant impacts on quality of life and presents several treatment challenges.
- These challenges are often expressed through the medium of social media, the analysis of which provides unique insights and perspectives on the condition from the point of view of both patients and health professionals.
- Paediatric dentists need to understand these perspectives in order to provide effective and holistic care to their patients.

## 1 INTRODUCTION

Amelogenesis imperfecta (AI) is a rare inherited disorder with a global prevalence of 1:700 to 1:14 000.^1^ The normal mechanism of enamel development is genetically altered in AI, which results in structural and visual changes to the enamel of both dentitions.^2^, ^3^, ^4^ Common AI features include pitted, grooved and discoloured teeth, microdontia, hypodontia, sensitivity and breakdown of teeth prior to or following eruption.^1^, ^5^, ^6^

Research has found a significant negative impact on individuals with AI.^7^, ^8^, ^9^, ^10^ Reduced quality of life is associated with the physical and psychological effects of the condition, as well as the impacts on social interaction, education and dental experience.^7^, ^8^, ^10^ Parents report adverse psychological impacts from AI, as well as negative dental experiences in relation to their child's care.^9^

Evidence‐based dental research to determine the most successful treatment options for effective management of AI is being conducted to aid the development of globally recognised guidelines for AI management. To manage AI effectively an interdisciplinary approach is advocated, including paediatric dental professionals and orthodontists in childhood then restorative specialists in adulthood.^2^, ^11^ Treatment options depend on the severity and classification of AI and include prevention, stabilisation, composite restorations, tooth whitening, microabrasion, conventional crowns, porcelain veneers and implants.^2^, ^11^, ^12^ The evidence collated from a review of current research suggests that indirect ceramic restorations could be the restorative treatment of choice for many patients with AI.^12^, ^13^, ^14^ There are, however, currently no globally recognised guidelines for AI management.

The clinical burden of care for young people and their families is significant. Lafferty et al. evidenced that multiple treatment episodes are necessary in the care of people with AI, with patients and their families often travelling significant distances to appointments.^15^ This research suggests specialist care constitutes a significant but worthwhile investment to patients and their families.

Social media encompasses a diverse range of platforms where users can interact and share content. Healthcare professionals use social media in a professional capacity to engage patients and to seek and share information.^16^ Individuals have engaged with social media to meet needs that cannot or are not being addressed by healthcare professionals, particularly an individual's need for support.^17^ Although this engagement can benefit patient relationships with healthcare professionals, for example, through improved communication and patient involvement in decision‐making, it can also present a challenge to healthcare professionals.^17^ Issues with continuity of care as a result of patients moving between clinicians and negative patient‐dentist interactions due to information obtained from social media are examples of such challenges.^17^ Research exploring the use of social media for a range of both general^18^ and dental conditions^19^, ^20^, ^21^ has demonstrated unique insights into the experience and needs of affected individuals. Given its impact and relative rarity, AI is likely to draw social media attention, the analysis of which will further our understanding of the issues for those managing and affected by the condition. This study used existing social media posts to examine how individuals and dental professionals share their experience of AI.

### 1.1 Aim

The aim of this study was to explore how public and dental professionals use social media to discuss amelogenesis imperfecta.

## 2 MATERIALS AND METHODS

This was a cross‐sectional study consisting of a systematic search of social media platforms to identify public and dental professionals' posts relating to AI, followed by a qualitative analysis of the word content of relevant posts. The search and analysis strategy for the study was adapted from previously reported methods.^19^ Ethics approval for this study was granted by the University of Leeds Dental Student Ethics Committee (19/10/2019). The Standards for Reporting Qualitative Research^22^ were followed in the preparation of this manuscript.

The two groups of social media users of interest for this study were as follows:

- Public: Any individual with personal experience of AI. This may be someone with AI or a family member or friend of someone with AI.
- Dental professionals: Dental professionals providing dental care for people with AI. This includes general dental professionals and specialists.

### 2.1 Method

#### 2.1.1 Scoping searches

A list containing the most commonly used social media platforms in the United Kingdom^23^ was consulted to identify suitable platforms for the study. The following social media platforms were excluded:

- Those using solely audio or video content (YouTube, Tik Tok and Vimeo);
- Those primarily being used for professional networking (LinkedIn and F6S);
- Those used for personal communication (WhatsApp, Skype and Snapchat); and
- Those with irrelevant content (Foursquare and Crunchbase).

Eighteen potential social media platforms were identified. After application of the exclusion criteria, eight were selected: Facebook, Instagram, Twitter, Tumblr, Reddit, Pinterest, Flickr and Medium.

Scoping searches were performed for each platform to identify which search terms would return the highest number of relevant posts. A preliminary search assessed the key words ‘amelogenesis imperfecta’, ‘amelogenesis’, ‘enamel disorder’, ‘enamel disorders’, ‘enamel defect’ and ‘enamel defects’. A substantial number of results from the search terms other than ‘amelogenesis imperfecta’ contained content that was not relevant, whereas using only ‘amelogenesis imperfecta’ did not result in any missed posts. As a result, only the term ‘amelogenesis imperfecta’ was used for all searches.

Social media platforms can be viewed according to various categories (eg, most recent and most relevant), so the category was selected for each social media platform to obtain the greatest number of recent and relevant results. The approximate quantity of results returned from each platform was assessed in the scoping searches to inform the setting of appropriate limits, where necessary, to ensure the volume of posts to be analysed was acceptable. The final search strategy is outlined in Table 1.

**TABLE 1:** Search strategy for social media sites

Platforms	Search terms	Category selected for viewing results	Limitations on search
Facebook	amelogenesis imperfecta	Posts	First 150 posts
Instagram	amelogenesis imperfecta	Most recent	First 150 posts
Twitter	amelogenesis imperfecta	Latest	First 150 posts
Tumblr	amelogenesis imperfecta	Recent	None
Reddit	amelogenesis imperfecta	Posts	None
Pinterest	amelogenesis imperfecta	n/a	None
Flickr	amelogenesis imperfecta	Photos	None
Medium	amelogenesis imperfecta	Stories	None

#### 2.1.2 Data acquisition

The definitive searches were conducted by two researchers. All social media posts identified in the searches were indexed into Microsoft Excel (v16). Each post was given an identification tag using letters to indicate to the social media platform and sequential numbering (eg, Tw12). For each post, standardised information was extracted (Table 2). Category codes were developed to aid assessment of eligibility for inclusion of the post in the study.

**TABLE 2:** Standardised data extraction for all social media posts identified in searches

	Categories	Coding (*eligible for content analysis)
1	Identifier	—
2	Initials of reviewer	—
3	Date of search	—
4	Social media platform	—
5	Search term inputted	—
6	Date of post	—
7	Language of post	English*/Non‐English
8	Whether the content was related to humans	Human*/Non‐human
9	Type of user	Public*/Dental professional*/Not evident/ Other
10	Publicly available information about the user (gender, age, geographic location)	—
11	Sufficient or insufficient English language to allow analysis	Sufficient*/Insufficient/Not applicable*
12	Post contains words other than solely the term ‘amelogenesis imperfecta’	Yes*/No
12	Post relevant to amelogenesis imperfecta	Relevant*/Not relevant
13	Word content of the post	—
14	Other content within the post (photographs, links)	—
15	Number of comments	—

#### 2.1.3 Selection of relevant posts

Each post was assessed against predefined eligibility criteria by two reviewers independently (Table 3). Posts that did not meet the inclusion criteria in any category were excluded with the reason recorded. Eligible posts were submitted for analysis of word content only; it was outside the scope of this study to analyse images and non‐text content.

**TABLE 3:** Eligibility criteria for inclusion of post in content analysis

	Inclusion criteria	Exclusion criteria
Type of content	Word content	Non‐word content
	English‐language posts or posts containing English and another language	Non‐English‐language posts
	Sufficient content to allow analysis Adequate number of words	Insufficient content to allow analysis Post where the only word is ‘amelogenesis imperfecta’
User	Posts by an individual member of the public with personal experience of AI	Posts by any other group
	Posts by a dental professional including general dentists and specialists	Posts where the type of user cannot be identified
Relevance of content	Posts related to humans	Posts that are not related to humans
	Posts that are relevant to amelogenesis imperfecta	Posts that are not relevant to amelogenesis imperfecta
	Original posts	Duplicate posts

#### 2.1.4 Data analysis

The word content of eligible posts was exported into Microsoft Word (v16). Data were analysed using the thematic approach described by Braun and Clark.^24^ The content of each post reviewed and coded to describe its meaning. The initial coding was discussed and revised by the whole research team until an agreed coding system was developed that was felt to accurately describe the data. Similar codes were then collated to develop themes using an iterative approach until a final analytic framework was agreed. Illustrative quotes were included with the results, but to fulfil ethical guidance, these were shortened to ensure it was not possible to identify the original post.

All researchers involved in the analysis were dentists, so it is important to acknowledge that their knowledge and pre‐existing beliefs may have influenced data interpretation. This was addressed by independent coding by individuals followed by a group discussion to identify and manage any assumptions that may have been made during analysis and interpretation.

## 3 RESULTS

Searches were conducted in December 2019. A total of 555 posts were identified, with approximately three quarters of posts arising from dental professionals and the majority being from Facebook, Instagram and Twitter. The application of the eligibility criteria resulted in a total of 144 posts for analysis (Table 4). Publicly available data about the user were restricted by privacy settings, limiting the scope for describing the sample. The majority of public posts originated from the United States of America, and the majority of users were female. Dental professionals were predominantly male, and posts were identified from every continent except for Antarctica.

**TABLE 4:** Results from the searches of the social media sites

Search results								
Social media platforms	Number of posts identified in search	Number of posts excluded by initial screening	Number of posts reviewed in detail			Number of posts eligible for analysis and included		
			Public	Dentist	Total	Public	Dentist	Total
Facebook	150	111	1	38	39	1	37	38
Instagram	150	77	3	70	73	3	50	53
Twitter	150	117	14	19	33	13	16	29
Tumblr	2	1	1	0	1	1	0	1
Reddit	22	3	18	1	19	17	1	18
Pinterest	69	66	0	3	3	0	3	3
Flickr	11	9	1	1	2	1	1	2
Medium	1	1	0	0	0	0	0	0
Total	555	385	38	132	170	36	108	144
Reason for exclusion								
Initial screening:	Type of content: 189 Type of user: 108 Relevance of content: 88		Detailed review:		Type of content: 0 Type of user: 0 Relevance of content: 26			

### 3.1 Use of social media

The public used Reddit and Twitter most commonly to discuss AI, including sharing information and experience, seeking information and seeking support. Reddit posts were generally longer in length, whereas several of the Twitter posts were replies to other users, which suggests Twitter can be used to have conversations regarding AI. Sharing experiences was the sole purpose of posts on both Instagram and Tumblr, whereas Facebook and Flickr were used exclusively for information sharing.

For dental professionals, the majority of posts on Instagram, Facebook and Pinterest were case reports of patients they had treated. Twitter was used to share information relating to AI. Flickr had a single post sharing information, whereas Reddit's only post was an advertisement. Most posts also contained images including pre‐ and post‐treatment clinical photography and radiographs. A small number of posts contained a link to an external website; these were outside the scope of the study and so were not analysed.

### 3.2 Public discussion of AI

Three main themes were identified to describe how individuals affected by AI discussed the condition and treatment: (1) experience of living with AI; (2) dental treatment for AI; (3) outcome from dental treatment (Table 5). These themes are summarised below with illustrative quotes.

**TABLE 5:** Description of thematic framework developed to describe the posts by the public

Theme	Subtheme	Topics	
Experience of AI	Understanding of AI	Diagnosis	Sharing information about their diagnosis; seeking information about the diagnosis
		Aetiology	Users evidenced a firm understanding of the aetiology of the condition, that is the genetic origin
		Dental health	Discussion regarding dental check‐ups and maintaining good oral hygiene
	Impact of AI	Physical	Reports of failure of dental treatment; negative impacts reported included pain when eating, tooth breaking, deterioration in appearance, decay and infection, and speech difficulties
		Psychosocial	Negative impact on smiling due to embarrassment; negative reaction from the public and dentists
		Coping and support	Seeking support or others with the condition; receiving support; sharing experiences; and coping strategies
Dental treatment	Experience of dental treatment	Self‐care	Oral hygiene; symptom management
		Dental treatment	Diagnosis; advice; pain management; prevention. Treatment: endodontic; restorative; oral surgery; prosthodontic; implants; orthodontic
		Experience of dentistry	Positive, for example smiling following the treatment. Negative, for example anxiety related to treatment, negative emotional response to comments of a dentist
	Barriers to treatment	Access and cost	Issues with insurance companies—continued high cost of treatment despite subsidisation. Interpretation of treatment being cosmetic vs routine
		Dentist inexperience	Challenges of inexperienced dentists Inexperience perceived to alter behaviour towards person with AI
		Perceived lack of options	Prosthodontic treatment No treatment to cure AI
Outcome from treatment	Expected or desired outcome	Physical	High expectations that may be challenging to achieve clinically Improved aesthetics Desire for a cure
		Psychosocial	Confidence; increase in smilin Stigma associated with dentures
	Actual outcome	Physical	Successful and unsuccessful outcomes
		Psychosocial	Increase in smiling; improvement in the quality of life

#### 3.2.1 Experience of living with AI

People discussed their experience of living with AI in terms of their understanding of AI and the day‐to‐day impact AI had on them.

Individuals affected by AI had a firm understanding of the aetiology of AI with numerous posts mentioning that the condition had a genetic origin. Individuals discussed being “*born without much enamel”* and having “*teeth without proper enamel*”.

The negative impact of AI was widely reported. Physical effects included pain when eating, teeth breaking, deterioration in appearance, decay and infection, and speech difficulties. Individuals described their teeth as “*ugly*”, “*yellow*”, “*messed up*” and having “*weird enamel*”. Psychosocial effects included embarrassment and low confidence as a consequence of bullying, a lack of understanding by other people and effects on the individual's behaviour, such as avoiding smiling.

People with AI shared their coping strategies, for example by making jokes about their condition before other people could mention it. There was evidence that people living with AI had support from their family. A post from a non‐affected individual discussed the psychosocial impact of AI on her husband, “*never seen him truly smile*”, and sought support on his behalf. Individuals often shared their own experiences as a way to gain and provide support. An account of an individual's experience with AI was followed by questions asking about others with similar problems and methods they use to cope.

#### 3.2.2 Dental treatment for AI

People with AI and their families discussed their experience of dental treatment for AI in terms of the type of treatment received, whether this was a positive or negative experience and, importantly, barriers to dental care. People with AI reported their own approach for self‐care and symptom management, for example using different types of toothbrush, using sensitive toothpaste and interdental cleaning.

The range of dental care received included dental professionals' diagnosis and advice; pain management such as the use of cold water and pain medication; preventative treatment; and interventions such as endodontic, restorative and prosthodontic treatment, oral surgery, provision of dental implants and orthodontic treatment.

Both positive and negative experiences of dental treatment were reported. A positive experience of dental treatment was illustrated in a post describing someone smiling for the first time following treatment. On the contrary, an individual reported their dentist linking the appearance of their teeth to poor oral hygiene rather than a genetic condition. Many people with AI reported dental anxiety, and an important finding was that people with AI felt there was a problem with dental professionals being inexperienced in recognising and treating AI, resulting in the dentist altering their behaviour and causing a negative impact on their care.

In most countries, the cost of dental treatment was identified as the main barrier to treatment. Several individuals highlighted problems with insurance companies, including high treatment costs despite subsidisation and companies' refusal to contribute to treatment costs on the basis that treatment was cosmetic rather than necessary. Three posts were identified that discussed raising money for treatment. A perceived lack of options was another barrier to treatment. For example, one person described the progressive deterioration of their teeth, with dentures eventually being the only option.

#### 3.2.3 Outcome from dental treatment

The outcome from dental treatment was described in terms of both expected and actual outcomes. There was evidence that people with AI had high expectations about the outcome from dental treatment that may be challenging to achieve. One individual discussed their desire for a “*teeth makeover*” and another their expectation of “*white perfect teeth*” following treatment. There was evidence that people felt a perceived stigma around dentures that would impact on their quality of life. The term “*old people*” was used by one person when discussing dentures. The desired outcome from treatment was related almost solely to confidence and willingness to smile.

Many individuals perceived the dental treatment they received had been unsuccessful in meeting their expectations. Those who had undergone dental treatment with which they were happy, however, reported better quality of life and an increase in smiling.

### 3.3 Dental professionals' discussion of AI

Dental professionals’ use of social media was most commonly sharing case reports (65.7% of posts), in addition to sharing information and seeking information (Table 6). Each of these uses of social media is described below.

**TABLE 6:** Description of thematic framework developed to describe the posts by dental professionals

Theme	Subtheme	Topics	
Case reports	Untreated AI	Background to AI	Diagnosis and, in some cases, also a differential diagnosis. Classification of types of AI Identification as a congenital disorder Inaccurate diagnoses including enamel hypoplasia and dentinogenesis imperfecta
		Presentation	Clinical features—discoloured, sensitive teeth Effect of AI—teeth prone to deterioration and caries with subsequent impact on the quality of life
	Treatment	Types of treatment	Variable treatment time of 3 months to 5 years. Various treatment modalities Provision of care by MDT Treatment recognised as challenging
		Outcome	Before and after photographs to demonstrate outcome Providing a life‐changing smile
		Advertising	Practice contact details
Sharing information	AI	Diagnosis	Use of scientific terminology Classification of 3–17 types of AI
		Aetiology	AI attributed to enamel protein mutations Suggestion that antibiotics in the first trimester cause AI
		Presentation	Complications from AI included increased caries risk, erosion, attrition, dentine hypersensitivity, fracture, gingival hyperplasia and early tooth loss
		Prognosis	Improved by treatment
	Management	Treatment options	Full coronal coverage to protect from dentine hypersensitivity and caries Contraindication of onlays/inlays due to the weak bond to enamel
		Provision of care	Early diagnosis and MDT care essential Advertisements for treatment
Seeking information	AI management	Clinical	Advice sought for managing challenging cases
		Research	Aetiology
	Professional development	Conference	Discussions relating to aetiology, management and improving prognosis for AI

#### 3.3.1 Case reports

Case reports, primarily shared on Instagram and Facebook and described in layman's terms, typically began with a definition of AI, then described the classification and clinical presentation. Other differential diagnoses included enamel hypoplasia and dentinogenesis imperfecta. Dental professionals identified AI as a congenital disorder and highlighted the increased caries risk. The main symptoms of the cases were reported to be discoloured and sensitive teeth that rapidly deteriorate, and dental professionals also reported psychosocial effects such as decreased confidence, which led to people seeking treatment before big life events, such as starting university.

Management of cases was largely multidisciplinary, and dental professionals acknowledged it was often challenging. Treatment included composite restorations, ceramic crowns and implants. Treatment duration ranged from 3 months to 5 years. Case reports were used as an opportunity to share the positive experience of providing a life‐changing smile, with emphasis on the patient's positive reaction following treatment.

#### 3.3.2 Sharing information

In contrast to case reports that were mainly in layman's terms, information‐sharing posts were generally longer with more scientific terminology. Many of the descriptions of clinical presentation used the same sentence but failed to provide a source for this information. Furthermore, different classifications were given but without clarity about which classification system was being cited. Aetiology was reported to be a result of enamel protein mutations; incorrect aetiological factors, however, were also stated, such as antibiotics in the first trimester. Reported sequelae of AI included increased caries risk, erosion, attrition, dentine hypersensitivity, fracture, gingival hyperplasia and early tooth loss.

Early diagnosis and multidisciplinary management were deemed essential to both patients' psychological well‐being and the quality of dental care. Full coronal coverage to protect from dentine hypersensitivity and caries was discussed, as well as the contraindication of onlays/inlays due to the weak bond to enamel.

#### 3.3.3 Seeking information

Posts on Twitter were predominantly related to current research or the British Society of Paediatric Dentistry Conference where aetiology, management and improving prognosis for AI were discussed. Advice was sought for difficult cases, and one advert for a pro bono full mouth reconstruction was identified. One clinician asked for assistance from others in completing a case to achieve ‘satisfactory results’.

## 4 DISCUSSION

This is the first study to investigate the use of social media by individuals affected by AI and dental professionals. The main purposes of social media use were to share and seek information, share experiences, seek support and receive support. The findings support studies in other areas of health care, which found that healthcare workers use social media to seek and share information^16^ and that patients incorporate social media use alongside their access to conventional health care.^17^ The identified themes were consistent with those in previous research, which suggested patients interact with social media to gain support from others with the aim of establishing positive social and psychological changes, and to evaluate their experiences against the experiences of other users.^17^ The same authors, however, suggested that patients use social media to discuss feelings in an environment that is not restricted by consideration of the thoughts of close relations,^17^ which was not a finding from this study.

Social media posts by those affected by AI demonstrated the significant negative impacts of AI on several aspects of daily life, which is consistent with evidence obtained from several other studies.^7^, ^8^, ^25^ Individuals shared their experience of AI, including the physical and social impact and their coping strategy. It appears social media provides a space for individuals to branch out and seek support from a wider group of people, which may be of particular benefit to individuals who do not know anyone else with AI. It also allows others with pre‐existing support, such as family members, to expand their support group and potentially gain a different form of support.

Information needs of individuals were identified, and these were largely related to uncertainty about diagnosis and treatment options. This suggests there is a demand for better quality information, information from a different perspective, or for an opportunity to clarify information with others to improve understanding. Seeking health‐related information through social media has been demonstrated in other studies,^19^, ^26^ which highlights a general issue in delivering relevant and accurate health information.

Several posts included inaccurate information, highlighting the challenges faced by the public when seeking information about AI and a potential lack of professional knowledge when trying to diagnose and treat AI. Recognition and correct citation of the different classifications for AI was lacking, demonstrating poor awareness of the complexities of AI and the need for clear, accurate information. In general, there was good understanding that the cause of the condition was genetic but no indication of the variety of individual genes that can be responsible. Identification of individual genes^27^ will significantly contribute to a more precise characterisation of phenotype reducing inconsistency and error both in clinical practice and, by extension, in social media. The current sharing of incorrect information on social media is potentially harmful, adding to uncertainty, anxiety and seeking inappropriate treatment. The ever‐changing nature of social media means information posted may quickly become outdated and misinformation can spread quickly.

The primary barrier to care mentioned by people with AI was the substantial cost of treatment, with and without insurance. People living with AI reported the misconception by insurance companies that AI treatment is cosmetic, rather than a necessary treatment. Other studies also found cost to be a barrier in the treatment of AI.^28^, ^29^, ^30^, ^31^ A further identified challenge for people with AI was their own dentist's inexperience of AI diagnosis and management. Other studies have demonstrated similar difficulties for patients with AI and their parents, who have struggled to discuss their condition with dental professionals.^8^, ^9^, ^10^

Case reports and posts sharing information formed the bulk of posts from dental professionals reflecting the rarity of the condition and the subsequent challenges in determining the best treatment. There are currently no local or globally recognised guidelines or gold standard for treating AI, which presents a challenge to those who see AI only rarely and will not have the clinical experience to inform decision‐making. By sharing information and examples of completed cases, dental professionals are attempting to increase the availability of information and support colleagues with less experience in managing AI; the reliability of information, however, is often questionable and this is compounded by inadequate source citation.

Sharing case reports does present several ethical and medico‐legal issues. Often confirmation that informed consent was obtained for the sharing of patient information on social media is absent. Furthermore, following upload to social media, the ownership rights to the clinical images and the rights of other users or the platform to use these clinical images is unclear. Sharing case reports potentially undermines the principles of evidence‐based dentistry because case reports may be biased if dental professionals only present their successful outcomes. This may cause dental professionals to feel inadequate in their own treatment or give patients unrealistic expectations of potential outcomes that treatment can achieve. Case reports predominately used layman's terms, indicating that these posts were not solely targeting dental professionals and it is possible the actual purpose was self‐promotion.

The posts seeking information identify there is a need for better information. This raises the question of who is responsible and accountable for providing up‐to‐date, evidence‐based information on platforms such as social media.

The utilisation of social media as a research tool offers a different perspective on the experiences of individuals affected by AI. Information obtained from social media may potentially be less prone to responder bias than other techniques, as users are posting their real‐world experience without any presumption about how it will be viewed. There are also limitations to social media: the sample does not represent the views and experiences of all individuals affected by AI; social media provides a snapshot in time and may not show the evolving views of users; only publicly available data is analysed, so this can introduce bias; dental professionals are likely to share cases that show their best work and are unlikely to discuss difficult experiences or poor outcomes candidly on public groups; and there is scope for misinterpreting information obtained from social media. Ideally, future use of social media as a research resource should include a stage in which the analysis and interpretation of information is reviewed by the individuals who posted.

Analysis of qualitative data can be prone to the biases of those interpreting the data. This was addressed by separate independent coding by the four authors, then review and discussion as a group to develop the analytic framework. The inclusion of a non‐dentist in the data analysis process may have been beneficial to counter the potential bias from a research team comprised solely of dentists; this was, however, not feasible within this study.

### 4.1 Implications and future research

Currently, there are no specific organisations to support people with AI, but the development of a recognised body, such as the Cleft Lip and Palate Association (CLAPA) or the Ectodermal Dysplasia Society, may be useful for both providing reliable, high‐quality information and providing peer support. Social media is potentially a valuable and accessible tool for sharing information and experiences, but this needs to be managed to prevent misinformation and to ensure individuals’ privacy is respected. Consideration needs to be given to whether advertising and ethical standards are being adhered to and who is responsible for the quality assurance of information. Research into the effectiveness of interventions to reduce barriers to care, such as those arising from insurance companies' misconceptions about AI, and alternative pathways for delivering care to overcome dental professionals' inexperience with diagnosis and treatment, may be helpful.

- Individuals affected by AI indicate there is a need for widespread support and better distribution of reliable information.
- The sharing of case reports by dental professionals suggests there are challenges in recognising AI and determining appropriate treatment options for rare conditions such as AI.
- There is potential for dental professionals to use social media in a more organised way to inform and support people with AI and to share information and learning with peers. Consideration needs to be given to patient confidentiality and privacy and the quality of information that is shared.

## AUTHOR CONTRIBUTIONS

RB and SB conceived the idea for the study; VH and SM collected the data; VH, SM, RB and SB analysed the data; and VH led the writing with contributions and full review by SM, RB and SB.

## FUNDING INFORMATION

None.

## CONFLICT OF INTEREST

VH, SM, RB and SB have no conflicts of interest.

## Data Availability

The data that support the findings of this study are available from the corresponding author upon reasonable request.
